# A beginner’s guide to radiation damage

**DOI:** 10.1107/S0909049509004361

**Published:** 2009-02-25

**Authors:** James M. Holton

**Affiliations:** a Department of Biochemistry and Biophysics, University of California, San Francisco, CA 94158-2330, USA; b Advanced Light Source, Lawrence Berkeley National Laboratory, Berkeley, CA 94720, USA

**Keywords:** radiation damage, minimum crystal size, protein macromolecular crystallography, dose doubling, radioprotectant, data collection strategy

## Abstract

Many advances in the understanding of radiation damage to protein crystals, particularly at cryogenic temperatures, have been made in recent years, but with this comes an expanding literature, and, to the new breed of protein crystallographer who is not really interested in X-ray physics or radiation chemistry but just wants to solve a biologically relevant structure, the technical nature and breadth of this literature can be daunting. The purpose of this paper is to serve as a rough guide to radiation damage issues, and to provide references to the more exacting and detailed work. No attempt has been made to report precise numbers (a factor of two is considered satisfactory), and, since there are aspects of radiation damage that are demonstrably unpredictable, the ‘worst case scenario’ as well as the ‘average crystal’ are discussed in terms of the practicalities of data collection.

## Abstract 2

Radiation damage considerations affecting data collection by more than a factor of two are summarized and damage avoidance strategies are suggested.

## 1. Introduction

It is not uncommon for radiation damage to prevent the solution of a structure. Diffraction spots can fade away before the data set is complete and heavy atoms sites can become disordered before sufficient anomalous signal is measured. The former is easy to detect by eye during data collection, but the latter is more insidious (Holton, 2007 ▶; Oliéric *et al.*, 2007 ▶). In either case, however, significant damage by the middle of the data set generally means that the data set is already ruined. What is more, the specific chemical changes induced by radiation damage can change the structure from the biologically relevant form, and this sometimes leads to wrong conclusions about function.

Radiation damage can be avoided by keeping the accumulated X-ray exposure short, but how short is short enough? How short is too short to solve the structure? Does it matter how bright the beam is? Is there anything that can be done during sample preparation or data collection that can mitigate or at least predict radiation damage?

There are several good reviews and broad reports on the field of radiation damage (Garman & McSweeney, 2007 ▶; Ravelli & Garman, 2006 ▶; Carugo & Carugo, 2005 ▶; Nave & Garman, 2005 ▶; Garman, 2003 ▶; Garman & Nave, 2002 ▶; Ravelli & McSweeney, 2000 ▶; Burmeister, 2000 ▶; Helliwell *et al.*, 1993 ▶), and the reader is referred to these works for comprehensive coverage of the field. What follows is a rough guide focusing on damage avoidance.

## 2. The factor of two

The cut-off of a factor of two for a radiation damage effect to be considered significant here was chosen because a factor of two in scattering power corresponds to a relatively small change in crystal size. Specifically, increasing all three linear dimensions of a crystal by 26% will double the volume of scattering matter (1.26^3^ = 2). Such a change in size appears to be a typical ‘error bar’ when examining crystals under a microscope, as most crystallographers will not distinguish between an 88 µm crystal and a 110 µm crystal, but the latter has twice the volume of the former and the number of photons a crystal will diffract before it is ‘dead’ is proportional to volume (see Appendix *A* [App appa]). Perhaps more attention should be paid to crystal size, but a factor of two can also be the uncertainty in X-ray beam intensity, especially if parameters such as the variability in storage ring current over a refill cycle are not taken into account. Increasing the exposure time by a factor of two will double the damage but increase the signal/noise ratio of the data by only ∼42% (1 *versus* 2^1/2^), an improvement which can be difficult to detect by inspecting a diffraction image, and in practice a factor of four in exposure time is generally needed to see new spots. A factor of two is also roughly the difference between collecting one wavelength or two (all other things being equal, such as the per-image exposure time), collecting the inverse beam wedge or not, and also the difference between collecting from one crystal or merging data from two crystals. So, a factor of two roughly corresponds to the decision thresholds that must be faced in data collection strategy.

## 3. What is a MGy?

The flux density (photons µm^−2^ s^−1^) of current synchrotron X-ray beams varies by a factor of ten thousand (Holton, 2008 ▶; Kuller *et al.*, 2002 ▶; http://biosync.rcsb.org/), so describing radiation damage in terms of ‘frames’ is not useful when trying to apply strategies learned using one beamline at another. A more transferrable unit is needed.

The Gy (J kg^−1^) is the SI unit of dose, which is the amount of energy absorbed by something per unit of mass, and protein crystals are typically given millions of Gy, so the MGy is a convenient unit. Obviously, the extent of radiation damage will depend on the number of photons absorbed, but it is not so much the photons themselves as the energy they carry that drives the chemical reactions of damage (Newton, 1963 ▶; Myers, 1973 ▶; Box, 1977 ▶), so it is most relevant and useful to describe damage in terms of dose. Note that fluence (incident photons µm^−2^) is sometimes incorrectly referred to as a dose, but the SI definition of dose is absorbed energy, not incident energy, and the correct meaning of the word ‘dose’ will be used here.

The relationship between fluence and dose depends on the X-ray wavelength and the atomic composition of the sample (see §6), but typically only a tiny fraction of the X-ray beam is absorbed by a protein crystal (usually ∼2%) so dose is generally independent of crystal size and directly proportional to fluence (incident photons µm^−2^). Typically, this ‘dose ratio’ (*k*_dose_) is ∼2000 photons µm^−2^ Gy^−1^. That is, a dose of 1 MGy will be deposited in a metal-free crystal after 20 s in a 100 µm × 100 µm beam of 1 Å X-rays with a flux of 10^12^ photons s^−1^. Note that if the crystal is bigger than the beam, then the dose to the part exposed to the beam will be proportional to incident photons µm^−2^ (see §7).

Neglecting the crystal thickness does introduce a small error. The actual dose will always be a little less than that given by *k*_dose_, but the error introduced is less than a factor of two as long as the crystal is thinner than the attenuation depth of the X-rays, which is 3600 µm in the above case. The error is less than 5% if the crystal is smaller than 370 µm thick.

The X-ray wavelength has a strong effect on *k*_dose_ and the exact dependence can be complicated (Hubbell, 2006 ▶; Seltzer, 1993 ▶). However, the empirical formula
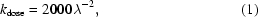
where *k*_dose_ is the dose ratio (photons µm^−2^ Gy^−1^) and λ is the X-ray wavelength (Å), is accurate to within 15% for 0.5 Å < λ < 3 Å. In fact, the simple assumption *k*_dose_ = 2000 photons µm^−2^ Gy^−1^ is accurate to within a factor of two for wavelengths between 1.1 and 0.9 Å. However, equation (1) assumes that no heavy (heavier than sulfur) atoms are in the crystal or solvent channels, and it may be off by much more than a factor of two if the heavy-atom concentration in the crystal is greater than ∼100 m*M* (see §6 and Table 1 ▶). For accurate determination of *k*_dose_ for an arbitrary wavelength and crystal formulation, use *RADDOSE* (Murray *et al.*, 2004 ▶, 2005 ▶; Paithankar *et al.*, 2009 ▶), but, even if *k*_dose_ is uncertain, the lifetime (in seconds) of crystals with given elemental composition at a given wavelength will always be inversely proportional to flux density (photons µm^−2^ s^−1^) when moving from one beamline to another, or as a given beamline is attenuated. Thus it is important to know the beamline flux (photons s^−1^) as well as the size of the beam at the crystal (µm^2^) (see §11).

It is also very important to remember that, like dose, diffracted intensities are proportional to fluence (photons µm^−2^) and have a rough λ^2^ wavelength dependence, but unlike dose they are relatively insensitive to heavy atom content. That is, for a given exposure time at a given X-ray wavelength, the amount of information obtained will depend on how many photons were thrown at the crystal, but the amount of damage inflicted will depend on how many were absorbed. Therefore, at some fixed wavelength, the value of *k*_dose_ is a good indicator of how much data a crystal will yield in its useful life relative to another crystal of the same size and type but different heavy atom content (see §6). A lower *k*_dose_ is better.

## 4. There are two kinds of radiation damage: global and specific

Irradiated protein crystals suffer an overall loss of resolution as high-angle spots fade away which is referred to as global damage. There are also specific chemical changes that can be seen in the electron density maps, such as side chains popping off. Specific damage can be up to ∼60 times faster than global damage (see below), but the good news is that at cryogenic temperatures the global damage ‘rate’ appears to be essentially the same for every protein crystal, once ‘lifetime’ has been normalized to dose (Garman & McSweeney, 2007 ▶; Owen *et al.*, 2006 ▶; Leiros *et al.*, 2006 ▶; Sliz *et al.*, 2003 ▶). In fact, the term ‘damage rate’ is something of a misnomer since the words ‘rate’ and ‘lifetime’ imply progression with time but the fundamental coordinate of cryogenic damage is dose. For this reason we introduce the term ‘lifedose’ to refer to the amount of dose a crystal can endure, and the word ‘lifetime’ will be used to indicate time.

Owen *et al.* (2006 ▶) recommended a general maximum tolerable dose (lifedose) of 30 MGy but noted there was also some resolution dependence to this as high-angle spots faded first. In fact, there is a remarkably linear relationship between scaling *B* factor and dose (Kmetko *et al.*, 2006 ▶; Borek *et al.*, 2007 ▶), but Howells *et al.* (2005 ▶) proposed a resolution-dependent dose limit criterion of 10 MGy per Å of resolution. For example, if a resolution of 3 Å is desired, the Howells criterion suggests a lifedose of 30 MGy. Since most of the dose limits used to derive this criterion used spot fading to half intensity as the indication of a dose limit, the fading of a spot at a given resolution can be supposed to follow an exponential decay,
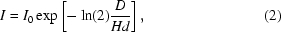
where *I* is the radiation-damaged spot intensity, *I*_0_ is the spot intensity at zero dose, ln(2) is the natural log of two (∼0.7), *D* is the dose in MGy, *d* is the *d*-spacing in Å and *H* is Howells *et al.* (2005 ▶) criterion (10 MGy Å^−1^). Note that, in equation (2), *I* = 0.5*I*_0_ when *D* in MGy is ten times the *d*-spacing in Å. This is not exactly the definition of *d* given by Howells *et al.* (2005 ▶), but equation (2) agrees remarkably well with recent damage studies. For example, applying equation (2) to the square structure factors of apoferritin [Protein Data Bank (PDB) ID: 2clu] results in a fairly linear fall-off of total intensity with dose that reaches half intensity at 42 MGy (not shown) which is consistent with 43 ± 3 MGy observed at half total intensity by Owen *et al.* (2006 ▶). In addition, scaling these same exponentially modified apoferritin data to unmodified intensities with *SCALEIT* (Howell & Smith, 1992 ▶) results in a best-fit relative *B* factor that increases linearly with dose having a slope of 1.3 *B*-factor units per MGy (B MGy^−1^), which is identical to the slope reported by Kmetko *et al.* (2006 ▶) for their apoferritin observations. Application of this same resolution-dependent spot-fading rate to lysozyme data (PDB ID: 2blx) reproduces the 1.03 B MGy^−1^ that Kmetko *et al.* (2006 ▶) reported for lysozyme, and thus explains the apparent protein-to-protein variability they observed. It is worthwhile noting that the sum of all diffracted intensities reduces by half if one applies a *B* factor of 15 to the apoferritin data, but this corresponds to a dose of 11 MGy using the slope 1.3 B MGy^−1^, a result that would be inconsistent with those of Owen *et al.* (2006 ▶) if damage manifested as a simple *B* factor. This is because the *B* factor has a resolution dependence of exp(−1/*d* ^2^), not the exp(−1/*d*) found by Howells *et al.* (2005 ▶). The Howells criterion of 10 MGy Å^−1^ therefore appears remarkably consistent with the observations of recent damage studies and is recommended as a good rule of thumb for predicting the lifedose of spots at a given *d*-spacing.

The bad news is that the rates of specific damage reactions are variable and depend on many factors including the folded structure of the protein (Holton, 2007 ▶), so there will probably never be a way to predict them before the structure is solved. This problem is exacerbated by the fact that ‘interesting’ parts of the molecule such as active sites, bound ligands and heavy-atom sites are particularly prone to specific damage. One might presume that this trend has anthropogenic origins because these are the parts of the protein where people spend the most time looking, but many systematic studies have now been carried out, and the trend does appear to be real (Burmeister, 2000 ▶; Ravelli & McSweeney, 2000 ▶; Weik *et al.*, 2000 ▶, 2001 ▶; Leiros *et al.*, 2001 ▶; O’Neill *et al.*, 2002 ▶; Nukaga *et al.*, 2003 ▶; Fuhrmann *et al.*, 2004 ▶; Carugo & Carugo, 2005 ▶; Dubnovitsky *et al.*, 2005 ▶; Roberts *et al.*, 2005 ▶; Yano *et al.*, 2005 ▶; Leiros *et al.*, 2006 ▶; Fioravanti *et al.*, 2007 ▶; Schiltz & Bricogne, 2007 ▶). The mechanism of site-specific radiosensitivity is still unclear. Several mechanisms for the high sensitivity of particular sites have been proposed, such as solvent accessibility (Burmeister, 2000 ▶; Weik *et al.*, 2002 ▶; Garman & Nave, 2002 ▶), the high X-ray cross sections of heavy atoms (refuted by Southworth-Davies *et al.*, 2007 ▶), chemical bond strain (Weik *et al.*, 2000 ▶; Fuhrmann *et al.*, 2004 ▶; Dubnovitsky *et al.*, 2005 ▶; Fioravanti *et al.*, 2007 ▶) and electrostatic field lines (Holton, 2007 ▶). For each of these models, there is both evidence and counter-examples, and the reader is referred to the above references for details. It will suffice here to say that there are probably at least two different radiochemical mechanisms at work, and the lifedose of any given site depends on its location in the structure.

Despite this inherent unpredictability, there are ‘world records’ for lowest lifedose of specific damage reactions: 2 MGy for selenomethionine (Holton, 2007 ▶), 0.5 MGy for bromouracil (Oliéric *et al.*, 2007 ▶) and as little as ∼0.3 MGy for the metalloprotein putidaredoxin (Corbett *et al.*, 2007 ▶), and these ‘worst case scenarios’ can be used when planning data collection (see §11).

## 5. Crystals are killed by photons µm^−2^, not time

One of the most remarkable findings about cryogenically cooled protein crystals is that global damage is proportional to dose, but not how fast that dose was delivered: the ‘dose rate’ (Garman & McSweeney, 2007 ▶; Sliz *et al.*, 2003 ▶; Leiros *et al.*, 2006 ▶; Owen *et al.*, 2006 ▶). This is certainly not the case at room temperature, where the extent of damage inflicted by a given dose does depend on the dose rate (Blake & Phillips, 1962 ▶; Southworth-Davies *et al.*, 2007 ▶), varies from protein to protein and even continues damaging the crystal after the X-rays have been turned off (Blundell & Johnson, 1976 ▶). However, unless stated otherwise, the discussion in this paper is about damage at cryogenic temperatures.

The exact timescale (and indeed the nature) of the cryogenic global damage reaction is not clear, but it must be very fast to have no dose-rate dependence and a lack of any demonstrable ‘dark progression’. Whatever reactive species are generated by the beam, they must be consumed as fast as they are made, or their concentration would build up at high dose rates, saturating the downstream reactions. This means that as long as a partially ‘burnt’ crystal is stored properly (such as described by Owen *et al.*, 2004 ▶) it will stay at liquid-nitrogen temperatures and diffract to the same resolution at a later time, even when restored to the beamline months later (not shown).

Specific damage is more complicated because a dose-rate dependence has been demonstrated (Leiros *et al.*, 2006 ▶) and damaged species have also been observed spectroscopically to disappear with a time scale of minutes after the beam has been turned off (Weik *et al.*, 2002 ▶; Southworth-Davies & Garman, 2007 ▶; McGeehan *et al.*, 2009 ▶). Nevertheless, no reported dose-rate dependence has been more than a factor of two. That is, the accumulated dose required to inflict a given quantity of damage (measured as site occupancy, scaling *B* factor or total diffracted intensity) has not been shown to change by more than a factor of two as a result of changing the dose rate. Consequently, dose-rate dependence will be considered insignificant here, and the reader is referred to the above references to learn more about it.

A very important consequence of the dose-rate independence of radiation damage is that the quality of data that can be obtained from a crystal before it is ‘dead’ will not change no matter how rapidly the photons are applied (flux or photons s^−1^). This is because the data are derived from scattered photons and scattering is exactly proportional to fluence (photons µm^−2^), which has no dimensions of time. This fact can be found in any of the many good books describing the physics of X-ray diffraction, such as Blundell & Johnson (1976 ▶) or Drenth (1999 ▶), with rigorous proofs given by Woolfson (1970 ▶) or James (1962 ▶). There are other parameters of beam quality such as collimation, spectral purity, crossfire and flicker noise which can impact data quality in various ways that will not be covered here, but flux (photons s^−1^) by itself does not affect data quality until it is converted into fluence (photons µm^−2^). Therefore, since there appears to be no significant dose-rate dependence to damage, the extent of damage is also proportional to fluence (photons µm^−2^), and the data-to-damage ratio is independent of the time taken to collect the data.

In the future, it may eventually become possible to collect data on timescales faster than the chemical reaction rates involved in damage, which will introduce a beneficial dose-rate effect. Exactly what this timescale must be is not presently clear as the rate constants (and indeed the mechanisms) of these reactions are not known.

## 6. Beware of high atomic numbers

The lifetime of a crystal in a given X-ray beam depends on the elemental composition of the crystal and the solvent inside it. Unlike visible light, X-rays are absorbed by all of the electrons in an atom, and the cross section increases roughly as *Z* ^2.7^ where *Z* is the atomic number. This means that absorption goes up very steeply moving across and down the periodic table. For example, 600 m*M* NaI instead of 600 m*M* NaCl in solvent channels (assuming solvent is 50% of the total volume) will cut the lifetime of a crystal roughly in half (see Kmetko *et al.*, 2006 ▶), but, since absorption is proportional to concentration, lower concentrations of NaI are less of a problem. Note that we are discussing lifetime and not lifedose, as the latter is remarkably unaltered by heavy atom content.

Precise X-ray absorption cross sections have been tabulated (McMaster *et al.*, 1969 ▶; Hubbell, 1982 ▶, 2006 ▶), and Seltzer (1993 ▶) generated effective cross sections accounting for energy-loss mechanisms such as X-ray fluorescence (which is ∼40% in the case of Se atoms) and assumed no self-absorption of fluorescent X-rays. The Seltzer (1993 ▶) tabulations were used to produce Table 1 ▶ because the error introduced by neglecting self-absorption is usually quite small (much less than a factor of two), and this correction was discussed in detail by Paithankar *et al.* (2009 ▶).

Table 1 ▶ was also made with the assumption that protein crystals and pure water have the same absorption. Although the average X-ray cross section of protein atoms is lower than that of water, the higher density of protein (∼1.34 g cm^−3^) tends to make up the difference. As long as the sample is free of heavy atoms, the dose (energy absorbed per unit mass) deposited by a given beam of X-rays in a thin layer of protein crystal is within 5% of that deposited in a thin layer of pure water for all photon energies between 5 and 50 keV.

Using these assumptions, the concentration of a given element that will cut a protein crystal lifetime in half (by doubling *k*_dose_) may be calculated. This concentration is moles of the element per litre of sample, not the molarity of the solution in the solvent channels. For example, if we fill the solvent channels of a protein crystal with a solution containing 680 m*M* of selenium, then the total concentration will be roughly 340 m*M* (assuming 50% solvent content and that the selenium compound does not bind to the protein).

Consider the photon energy to be 12680 eV. The mass energy absorption cross section that Seltzer (1993 ▶) provided for water at this energy is 2.3 cm^2^ g^−1^, so exposing a thin layer of water to a fluence of 10^6^ photon cm^−2^ will deposit 2.9 × 10^10^ eV cm^−3^ (10^6^ photon cm^−2^ × 2.3 cm^2^ g^−1^ × 12680 eV photon^−1^ × 1.0 g cm^−3^) of energy. The mass energy absorption cross section of selenium at this energy is 87 cm^2^ g^−1^, so a gas of selenium atoms at 27 mg cm^−3^ will absorb the same amount of energy per unit volume as water (10^6^ photons cm^−2^ × 87 cm^2^ g^−1^ × 12680 eV photon^−1^ × 0.027 g cm^−3^ = 3.0 × 10^10^ eV cm^−3^). Therefore, an aqueous solution containing 27 mg cm^−3^ (340 m*M*) selenium will absorb roughly twice as much energy as pure water or a native protein crystal.

If we consider lighter atoms, then the volume occupied by the solute can become significant and must be taken into account. For example, 14 mol L^−1^ of sodium will absorb as much energy as an equal volume of water, but a 14 *M* aqueous solution of sodium will contain significantly less water per unit volume than pure water, and therefore absorb less than twice as many X-rays as pure water. If we assume that each sodium atom displaces one water molecule, then 19 *M* sodium is required to double the dose. Very light elements absorb fewer X-rays per atom than the water they displace, and adding lithium will actually reduce the total X-ray absorption. However, cutting the X-ray absorption in half requires displacing more than half of the water, or roughly 28 *M* Li, a practical impossibility.

Table 1 ▶ was calculated as above using X-rays at the Se edge (0.9793 Å) and these dose-doubling concentrations will be different at other wavelengths. As an extreme example, the dose-doubling concentration of Br is ∼1.2 *M* at 0.9793 Å (below the Br *K*-edge) but this will drop to 320 m*M* at 0.9193 Å (above the Br *K*-edge). To calculate exactly how a particular crystal composition will behave at a particular wavelength, use *RADDOSE* (Murray *et al.*, 2004 ▶, 2005 ▶; Paithankar *et al.*, 2009 ▶). However, to within a factor of two anything at less than ∼100 m*M* is ‘safe’. Note, however, that this does not mean 99 m*M* is the same as 0 m*M*; the effect is proportional to concentration.

## 7. The ‘spreading’ of radiation damage is ∼3 µm

Cryo-cooled protein crystals are solids, and this is evident if they are mounted with the wrong size cryo-tongs: the droplet containing the crystal can be crushed or shattered like any other glass. Since the viscosity of a glass is incredibly high, there can be no diffusion of radicals in the traditional sense (Douzou, 1977 ▶). That is, there is no mass transport except on geological timescales (thousands to millions of years). There is also not enough energy in available X-ray beams to heat a crystal by more than a few degrees (Snell *et al.*, 2007 ▶). This is not to say that massless reactive species cannot move as much as a few dozen angstroms or more in a glass, they can (Box, 1977 ▶; Petrik & Kimmel, 2003 ▶, 2004 ▶), but this is solid-state chemistry, which is very different from radiation chemistry in aqueous solution at room temperature (Zagórsky, 1999 ▶).

It is possible to shoot one part of the crystal so much that it expands physically and the strain induced by this expansion distorts the lattice of neighbouring regions of the crystal (not shown), but the bulk of data collected at the Advanced Light Source beamline 8.3.1 (MacDowell *et al.*, 2004 ▶) by ‘walking’ down needle crystals shows no signs of damage ‘spreading’ any more than a few micrometres, and a systematic study by Schulze-Briese *et al.* (2005 ▶) found that damage is indeed limited to the irradiated area. Since the range of a ∼10 keV photoelectron in organic matter is ∼3 µm (Cole, 1969 ▶), there is no physical reason to think that damage ‘travels’ any further than that. Conversely, half of the absorbed energy should escape a crystal which is smaller than ∼40% of the photoelectron range (Nave & Hill, 2005 ▶), but a practical demonstration of this effect has yet to appear in the literature.

## 8. Scavengers and radioprotectants

There are now seven molecules that have been reported to have a protective impact on specific radiation damage including ascorbate (Murray & Garman, 2002 ▶; O’Neill *et al.*, 2002 ▶; Betts, 2004 ▶; Southworth-Davies & Garman, 2007 ▶; Holton, 2007 ▶), nicotinic acid and DTNB (5,5′-dithiobis-2-nitrobenzoic acid) (Kauffmann *et al.*, 2006 ▶), nitrate ion (Borek *et al.*, 2007 ▶; Holton, 2007 ▶) and 1,4-benzoquinone, TEMP (2,2,6,6-tetramethyl-4-piperidone) and DTT (dithiothreitol) (Southworth-Davies & Garman, 2007 ▶). These workers also found a much longer list of substances that have no effect.

As for global damage, Kauffmann *et al.* (2006 ▶) and Murray & Garman (2002 ▶) both reported a protective effect from the additives listed above. Murray & Garman (2002 ▶) did not claim more than a factor of two change in any of the metrics used, but Kauffmann *et al.* (2006 ▶) reported better than a factor of two impact on both global and specific damage. Unfortunately, the results reported by Kauffmann *et al.* (2006 ▶) were not normalized for dose nor for scattering power (see §10), so it is difficult to assess the impact of these additives on a transferrable scale. Nevertheless, if a crystal is found to tolerate the presence of any of these additives, it is advisable to try them.

## 9. Helium

At any temperature, using helium gas instead of nitrogen will reduce background scattering (Polentarutti *et al.*, 2004 ▶), and this effect is particularly prominent inside the ‘water ring’ (*d*-spacings between 3.8 Å and the beam stop) where helium scatters 1/49th as much as air. At the water ring and higher angles, the scattering from the sample is roughly equivalent to that of 1000 times its thickness of air, which tends to overwhelm air scatter unless the air path is more than 1000 times longer than the sample is thick. For example, a 3 cm air path is significant if the sample (crystal + cryosolvent) is 20 µm thick, but not if the sample is 200 µm thick. Some of the historical confusion about the benefits of helium arose from the need to de-convolute this effect, but the more recent studies have taken it into account. Nevertheless, from a data collection standpoint, the background reduction alone can be a good enough reason to use helium, even at 100 K.

As with radioprotectants, there have been plenty of negative results attempting to use temperatures lower than 100 K to reduce radiation damage and a few have even been published (Meitzner *et al.*, 2005 ▶), but there are now several reports of significant reduction of specific damage at temperatures from 7 to 40 K (Yano *et al.*, 2005 ▶; Grabolle *et al.*, 2006 ▶; Corbett *et al.*, 2007 ▶). All of these reports were reductions in active site damage of metalloproteins ranging from factors of two to a factor of 30 in Corbett *et al.*, but this does not mean that helium temperatures will not slow down other specific damage reactions.

As for global damage, there certainly are reports of a positive impact from helium cryostats (Teng & Moffat, 2002 ▶; Hanson *et al.*, 2002 ▶; Chinte *et al.*, 2007 ▶; Borek *et al.*, 2007 ▶; Meents *et al.*, 2007 ▶), but no reports thus far have claimed better than a factor of two in sample lifedose. Greater than a factor of two reductions in global damage has been reported in cryo-electron microscopy, but the benefits of helium are still controversial in this field (Massover, 2007 ▶; Glaeser, 2008 ▶).

## 10. The minimum crystal size to solve a structure

It was shown in §4 that diffraction spots from any protein crystal at a given *d*-spacing will fade with essentially the same lifedose, so the amount and quality of data that can be collected before a crystal ‘dies’ will depend on the initial scattering power of the crystal. For example, given a particular data-quality goal, crystals with larger unit cells will have to be bigger than those with smaller unit cells because spot intensity is proportional to the number of unit cells in the beam. The relationship between all the factors affecting scattering power and radiation damage is detailed in Appendix *A* [App appa], where the number of crystals of a given type and size needed to solve a structure is derived,
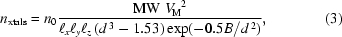
where *n*_xtals_ is the number of crystals required to solve the structure, *n*_0_ is the number of crystals required in a unitary reference case (see below and Table 2 ▶), ℓ_*xyz*_ is the crystal size in each direction (µm), MW is the molecular weight (kDa), *V*_M_ is the Matthews number (Å^3^ Da^−1^), *d* is the *d*-spacing of interest (Å) and *B* is the Wilson *B* factor (Å^2^). The crystal size, molecular weight, approximate *V*_M_ and resolution of interest are all readily available quantities to the crystallographer screening crystals, but it can be difficult to know the Wilson *B* factor *a priori*. A survey (not shown) of the PDB (Berman *et al.*, 2000 ▶) revealed a simple relationship between the resolution limit (*d*_min_) and the average atomic *B* factor of entries claiming that resolution limit,
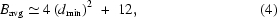
which holds in the range 1.5 Å < *d*_min_ < 4 Å. Whatever the reason behind this trend, *B*_avg_ is empirically a rough estimate of the Wilson *B* factor of a crystal that appears to diffract to *d*_min_ on initial screening diffraction images and this can be substituted into equation (3).

The value of the empirical ‘difficulty parameter’ *n*_0_ will depend on the type of experiment to be attempted, and it can be interpreted as the number of crystals required when the fraction in equation (3) is equal to 1.0, such as 5 × 5 × 5 µm lysozyme crystals with Wilson *B* = 21 and *d* = 2 Å. Experiments with higher data quality requirements (see below) will have larger values of *n*_0_, but improvements in methodology and equipment are expected to lower *n*_0_ for a given experiment.

The usefulness of equation (3) is demonstrated in Table 2 ▶. It can be seen that *n*_0_ for the goal of obtaining a complete native data set has been decreasing over time but has never been less than 3. Therefore, using *n*_0_ = 3 in equation (3) is a good way to gauge whether trying to obtain a complete data set is hopeless. That is, if plugging in the parameters of a crystal of interest and *n*_0_ = 3 results in *n*_xtals_ > 1, then obtaining a complete data set from a single crystal would be a record-setting feat. It is not recommended to try to set new records with projects that might require more than just a complete data set. That is, 90% complete data may be sufficient for molecular replacement and refinement to succeed, but the integrity of important chemical bonds can be questionable at *n*_0_ = 3.

A case in point is MAD/SAD structure solution, as it was noted in §4 that metal sites can break down up to 60 times faster than the observed decay of spot intensities. This implies that, for ‘safe’ structure determination by MAD or SAD, *n*_0_ should be increased ∼60-fold to *n*_0_ = 180. Covalent bonds in active sites and ligands can break rapidly as well, but these decay rates are currently not well characterized, so, if breaking a particular bond would change the conclusions derived from the structure, a recommended strategy is to use *n*_0_ ≃ 180. For example, the high value of *n*_0_ for the 3.5 Å structure of the ribosome (Schuwirth *et al.*, 2005 ▶ in Table 2 ▶) is due to the fact that care was taken to avoid any signs of radiation damage in those data (∼2 MGy per crystal) and that the dose ratio (*k*_dose_) of nucleic acids is about twice that of protein (owing to phosphorous content). In general, addition of any of the dose-doubling concentrations listed in Table 1 ▶ requires doubling the value of *n*_0_. This is because *n*_0_ is linear with dose.

Note that a value of *n*_xtals_ greater than 1 does not mean that the crystal should be discarded. Walking a small beam down a needle crystal to *n*_xtals_ different locations and merging the data will satisfy equation (3). Remember that the beam size will ‘cut off’ the effective crystal dimensions (see §7) and that should be reflected in the values of ℓ_*x*_, ℓ_*y*_ and ℓ_*z*_ because the product of these three numbers is the volume of scattering matter. For example, a 10 × 10 µm beam on a crystal 100 µm in all dimensions is equivalent to a crystal and beam which are 22 µm in all dimensions, because 10 × 10 × 100 = 10^4^ and 22 × 22 × 22 ≃ 10^4^. However, rotating a large crystal in a small beam will change the effective exposed volume as well (see below).

## 11. Summary and general strategy recommendations

Radiation damage begins with the first X-ray photon absorbed in the crystal, so data collection strategy must always be a balance between the data quality required and the amount of damage than can be tolerated. It is often the case that this balance cannot be struck with just one crystal and perhaps not even with many if the crystals are very small and very weakly diffracting. So, given a new and very precious protein crystal, how shall one proceed with data collection?

The first thing that must be known about a beamline or other X-ray source is the flux (ϕ) in photons s^−1^, and this can be obtained using a calibrated photodiode (Owen *et al.*, 2009 ▶). The next parameters to obtain are the dimensions of the beam at the crystal (ℓ_Hbeam_ and ℓ_Vbeam_), as these are needed to compute the flux density (photons µm^−2^ s^−1^). If these are not readily available, they can be obtained by exposing a small piece of silica glass (such as a cover slip) which will turn brown in the X-ray beam. If necessary, this darkened glass can be transferred to a microscope with a calibrated reticule for measurement of the beam size.

Once the flux density (photons µm^−2^ s^−1^) is known, all that remains is the dose ratio (*k*_dose_) which is usually 2000 photons µm^−2^ Gy^−1^, but can also be calculated more precisely for a given wavelength and chemical composition of the crystal using *RADDOSE* (Murray *et al.*, 2004 ▶, 2005 ▶; Paithankar *et al.*, 2009 ▶). Given all these, the maximum recommended shutter-open time a crystal will endure can be calculated,
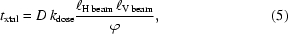
where *t*_xtal_ is the maximum shutter-open time for a data set (s), *D* is the expected lifedose of the crystal (Gy), *k*_dose_ is the dose ratio (∼2000 photons µm^−2^ Gy^−1^), ϕ is the beam flux (photons s^−1^), ℓ_Hbeam_ is the dimension of the X-ray beam spot along the spindle direction (µm) and ℓ_Vbeam_ is the dimension of the X-ray beam spot perpendicular to the spindle (µm). Some data collection strategy programs such as *BEST* (Bourenkov & Popov, 2006 ▶) can take *t*_xtal_ as input, but it is instructive to examine its meaning here. For example, if it is desired to collect a complete data set to 2 Å resolution, then we obtain *D* = 20 × 10^6^ Gy using the Howells criterion (see §4). Assuming the X-ray wavelength is ∼1 Å (see §3) and no heavy-atom concentration is above 100 m*M* (see §6), the *k*_dose_ given above will roughly apply, and, using a beam 100 µm high and 200 µm wide with flux 1 × 10^12^ photons s^−1^, we obtain *t*_xtal_ = 800 s. This means that a data set of 100 images should use a per-image exposure time of 8 s or less. A 3 Å data set, however, could use 12 s exposures.

Note that *t*_xtal_ is proportional to *d* *via* the Howells criterion, but this does not imply that shorter *t*_xtal_ will yield better resolution; rather, measuring high-angle spots must be done quickly because they will endure less dose than low-angle spots. Increasing flux (photons s^−1^) will also reduce *t*_xtal_, which means that the data can be collected faster, but the number of photons scattered into the detector before the spots fade away will not change. This is a consequence of the lack of a significant dose-rate dependence to radiation damage (see §5).

On general terms it is recommended to try to match the size of the beam to the size of the crystal as this optimizes the ratio between diffracted intensities and background scattering. However, this is not always possible and care must be taken when considering a mismatch in size between the beam and the crystal. Equation (5) is based on the assumption that the crystal is ‘bathed’ in the X-ray beam, and a crystal larger than the beam and rotating will complicate the dose calculation as ‘fresh’ and ‘partially burnt’ crystal matter moves in and out of the illuminated region. This process can be roughly accounted for by assuming that the average dose (total energy absorbed divided by the total mass that absorbed it) applies. The correction to equation (5) is then the cross-sectional area of the exposed region of the crystal (viewed down the spindle axis) divided by the average path length traversed by the beam through the crystal as it rotates. This correction is indeed geometrically involved, but a reasonable approximation to it (within 25%) is achieved by replacing ℓ_Vbeam_ in equation (5) with the geometric mean of the two crystal dimensions perpendicular to the spindle [(ℓ_*x*_ℓ_*y*_)^1/2^].

For example, consider a crystal that is a cylindrical rod 50 µm in diameter and 1 mm long with the long dimension oriented along the rotation axis. If this crystal is centred in a 10 × 10 µm beam and the data collection rotates it by 180°, then the exposed volume is a 50 µm-diameter disc, 10 µm thick (volume ≃ 20 × 10^3^ µm^3^), but the total energy absorbed is identical to that of a 10 × 10 × 50 µm exposed volume (5 × 10^3^ µm^3^) that did not rotate. The average dose (J kg^−1^) is therefore four times less than that assumed by equation (5) as written, and *t*_xtal_ will actually be four times longer. Replacing ℓ_Vbeam_ in equation (5) with (ℓ_*x*_ℓ_*y*_)^1/2^ = 50 µm is more accurate, but actually represents a 25% over-correction in this case. This over-correction is less if the exposed region of the crystal has a rectangular cross section (viewed down the spindle axis). In fact, it can be shown that the result obtained by numerically integrating the average dose to such a rectangular illuminated volume is quite similar to that obtained from equation (5) after replacing ℓ_Vbeam_ with (ℓ_*x*_ℓ_*y*_)^1/2^. The difference ranges between a 12% over-correction to a 25% under-correction when the ratio of the crystal edges (ℓ_*x*_:ℓ_*y*_) ranges from 1:1 to 10:1 (respectively).

If the beam profile is a ‘top hat’ with even illumination at every point (constant flux density or photons s^−1^ µm^−2^) then crystals smaller than the beam will still obey equation (5). However, if a small crystal is in a ‘hot’ central region of a larger beam, it will burn up faster than predicted by equation (5), since it is actually experiencing the flux density (photons s^−1^ µm^−2^) passing through its own cross-sectional area (looking down the beam). For example, a Gaussian beam profile will have a peak flux density that is 88% of the ‘average’ flux density assumed by entering the whole-beam flux (photons s^−1^) as ϕ and the two full width at half-maximum (FWHM) dimensions of the beam profile as ℓ_Hbeam_ and ℓ_Vbeam_ in equation (5). Similarly, a ‘round’ crystal that exactly matches the FWHM dimensions of a Gaussian beam will experience 50% of the whole-beam flux ϕ (photons s^−1^). Crystals in such beams can therefore be expected to last roughly 13% longer or twice as long as predicted by equation (5), respectively. This is not a radiation-protective effect of Gaussian beams, but merely an artefact of computing flux density.

It is noteworthy that equation (5) is still accurate to within a factor of two despite all these caveats (providing it is corrected for the large crystal rotating in a small beam case), but significantly more accurate sample lifetimes given different beam shapes and sample sizes can be obtained using *RADDOSE* (Murray *et al.*, 2004 ▶, 2005 ▶; Paithankar *et al.*, 2009 ▶).

The above example uses the Howells criterion for the lifedose of global damage, but if specific damage is a concern then the lifedose of the relevant reaction should be used. For example, selenomethionine sites have never been observed to be more than half-decayed after a dose of 2 MGy (Holton, 2007 ▶), and using this value for *D* in the example above we obtain a maximum per-image exposure time of 0.8 s. This is an example of why it is generally necessary to grow larger and higher-quality crystals for MAD/SAD structure solution than for a complete data set, as the former will diffract more photons into the detector for a given exposure time.

It will often be the case that a diffraction image taken with the exposure time recommended by equation (5) will have an unsatisfactory appearance, such as the absence of high-angle spots that were clearly evident on a longer exposure. This generally means that the crystal at hand will not yield enough data to meet the specified goals. The next step is therefore either to change the crystal or change the goals. For example, if it is decided that only 10 s exposures are satisfactory, but selenomethionine decay is a concern, then *t*_xtal_ = 80 s implies that only eight images can be collected before there is a danger that some of the sites have become disordered. However, some selenomethionine side chains are particularly hardy, so it is prudent to continue collecting data until the spots fade away with the caveat that only the first eight images may contain anomalous signal. There are two ways to address this caveat.

(i) If more than one crystal is available, then an advisable strategy is to combine the first ∼2 MGy worth of data from several crystals into a composite data set. In this case care must be taken to collect the early data from each crystal in different regions of reciprocal space. This technique was developed by Kendrew *et al.* (1960 ▶) and employed more recently by Facciotti *et al.* (2003 ▶).

(ii) If only one crystal is available, or non-isomorphism between crystals is problematic, then resolution must be sacrificed in order to obtain complete data. In this case, it is recommended to collect a complete data set with short exposures first (*i.e.* the 0.8 s exposures in the above example) and then keep collecting additional complete data sets, multiplying the exposure time by a factor of three each time. This protocol not only extends the effective dynamic range of the detector, but roughly doubles the overall signal/noise ratio with each new data set if everything is merged together in the end. Merging is recommended if there are no signs of damage. Signs of global damage are an increasing scaling *B* factor (Kmetko *et al.*, 2006 ▶; Borek *et al.*, 2007 ▶), and a reduced anomalous signal (*R*_anom_ or CC_anom_; Evans, 2006 ▶) is an excellent indicator of specific damage to metal sites. If rapid specific damage is evident, then merge the early and late data separately and treat them as RIP data (Ravelli *et al.*, 2003 ▶, 2005 ▶; Nanao *et al.*, 2005 ▶; Banumathi *et al.*, 2004 ▶; Zwart *et al.*, 2004 ▶). In this way, the same collection strategy can be used for two different structure-solving pathways: RIP will be appropriate if the damage was fast, and MAD/SAD if it was slow.

## Figures and Tables

**Table 1:** Dose-doubling concentration at 12680 eV/0.9793 Å (the Se edge) A protein crystal containing the indicated element at the concentration shown will absorb roughly twice as much energy (dose) as a metal-free protein crystal when exposed to an X-ray beam with photon energy 12680 eV. Bear in mind that the concentrations shown are in terms of moles of the indicated atom per unit volume of sample (see text). This calculation assumed that protein has roughly the same energy absorption as water and that one water molecule was replaced by each atom of the indicated element, which becomes important for high concentrations. Details of the calculation are explained in §6. The asterisk (*) on the Br entry is a reminder that the dose-doubling concentration of Br is high for 12680 eV, but drops to 320 m*M* at 13486 eV.

Na	19 *M*	As	350 m*M*
Mg	12 *M*	Se	340 m*M*
P	4 *M*	Br*	1.2 *M*
S	3 *M*	I	230 m*M*
Cl	2.5 *M*	Gd	110 m*M*
K	1.6 *M*	Ta	75 m*M*
Ca	1.3 *M*	Pt	100 m*M*
Fe	560 m*M*	Au	100 m*M*
Cu	430 m*M*	Hg	88 m*M*
Zn	400 m*M*	U	100 m*M*

**Table 2:** Experimental determinations of minimum crystal size This table lists values for *n*_0_ determined from the scattering power of crystals for which the minimum size required for a complete data set has been reported. The parameters listed in the first two rows are examples that both use the same value of *n*_0_ for equation (3) and demonstrate that the size requirement of different crystal types can still be governed by a single *n*_0_ parameter. Note that *n*_0_ appears to be restricted to a relatively small range when compared with the variety of molecular weights and crystal sizes shown, and that *n*_0_ has been decreasing over time, perhaps as instrumentation and algorithms have improved. Footnotes indicate derived parameters and an asterisk (*) indicates that equation (4) was used to estimate the Wilson *B* factor. A question mark (?) indicates that the parameter was not provided in the given reference, but a reasonable average value for protein crystals was substituted.

MW (kDa)	Resolution (Å)	*V*_M_ (Å^3^ Da^−1^)	Wilson *B* (Å^2^)	Crystal size (µm)	No. of crystals	*n*_0_	Reference
14	1.5	2.0	20	20	1	3.1	Example
100	2.5	2.4	40	15	1	3.1	Example
62†	1.9	2.4?	20*	30	13	130	Gonzalez & Nave (1994 ▶)
14	1.6	2.0	22*	35	1	25	Teng & Moffat (2000 ▶)
28	2.1	2.5	30	20	1	12	Glaeser *et al.* (2000 ▶)
24	2.0	2.5	22	5 × 30 × 30	5	9.8	Facciotti *et al.* (2003 ▶)
400	3.5	2.5	65*	20	1	9.3	Sliz *et al.* (2003 ▶)
28.6	1.98	1.58	11	5	2	5.2	Coulibaly *et al.* (2007 ▶)
0.8	1.3	1.5	10	1.5 × 1.5 × 5	3	3.7	Nelson *et al.* (2005 ▶),Sawaya *et al.* (2007 ▶)
78	2.65	3.06	56	16‡ × 5 × 5	4	3.6	Li *et al.* (2004 ▶)
73	3.4	3.67	69	5	13	3.2	Standfuss *et al.* (2007 ▶)
21	1.5	2.4	11.4	1 × 1 × 20	90	3.1	Moukhametzianov *et al.* (2008 ▶)
6000	3.46	3.4	70	70	17	180	Schuwirth *et al.* (2005 ▶)

† Estimated for 100 Å unit cell in *P*4_3_2_1_2 with *V*_M_ = 2.4.

‡ Taken from 400 µm^3^ illuminated volume quoted by Moukhametzianov *et al.* (2008 ▶) and 5 µm beam.

## Appendix A Derivation of equation (3)

The ratio of scattered to incident photons has been referred to as the scattering power of a crystal (Sliz *et al.*, 2003 ▶; Teng & Moffat, 2000 ▶), which is embodied in the classic formulae due to Darwin (1914 ▶), one of which is simplified here,

where *I*_spot_ is the integrated spot intensity, *V*_xtal_ is the volume of the crystal, *V*_cell_ is the volume of the crystal unit cell, *t* is the exposure time, *L* is the Lorentz factor, *P* is the polarization factor and *F* is the structure factor (equivalent electrons per unit cell). Although Darwin (1914 ▶) showed that diffraction strength is inversely proportional to the square of the unit-cell volume, the unit-cell volume is, in turn,

where MW is the molecular mass of the asymmetric unit (Da), *n*_symops_ is the number of symmetry operators in the space group and *V*_M_ is the Matthews number (∼2.4 Å^3^ Da^−1^; Matthews, 1968 ▶). In addition, Wilson (1949 ▶) showed that the average squared structure factor is proportional to the number of atoms in the unit cell,

where *f*_a_ is the average atomic structure factor of a protein atom (electrons), *B* is the average atomic *B* factor (Å^2^) and *d* is the *d*-spacing (resolution) of the spot of interest (Å). We will also assume that the full lifedose of the crystal is utilized, which means that the effective total accumulated exposure time (*t*) will be proportional to the *d*-spacing (*d*) of the spot because low-angle spots last longer (Howells *et al.*, 2005 ▶). In addition, higher symmetry crystals require less rotation range, so for any strategy that yields complete data the total number of photons delivered to a given unique *hkl* index will be proportional to the number of symmetry operators (*n*_symops_),

This effective exposure time is not the only parameter that varies with resolution. The atomic structure factor (*f*_a_), the Lorentz (Darwin, 1914 ▶; Blundell & Johnson, 1976 ▶; Lipson & Langford, 2006 ▶) and polarization (Azaroff, 1955 ▶; Kahn *et al.*, 1982 ▶; Drenth, 1999 ▶) factors all depend on the *d*-spacing of the spot. The average product of all these terms can be described by the empirical expression

Excluding a scale factor, this expression is accurate to within 10% error for *d*-spacing between 1.5 and 4.5 Å. Combining all the above expressions we arrive at an expression for the average attainable spot intensity at a given resolution,

We now decompose the total volume of scattering matter (*V*_xtal_) into a number of crystals (*n*_xtals_) with explicit dimensions (ℓ_*x*_, ℓ_*y*_ and ℓ_*z*_), and solve for the required number of crystals,

Assuming that some critical average spot intensity is required, we can replace the intensity term with an arbitrary proportionality constant (*n*_0_) to represent this requirement,

The *n*_0_ parameter may now be obtained empirically by plugging in the parameters from a previous experiment where the data were found to be sufficient to reach a given goal, such as a complete data set or solving a MAD structure. An accounting of the former is given in Table 2 ▶.
